# Microplastic fragments and microbeads in digestive tracts of planktivorous fish from urban coastal waters

**DOI:** 10.1038/srep34351

**Published:** 2016-09-30

**Authors:** Kosuke Tanaka, Hideshige Takada

**Affiliations:** 1 Laboratory of Organic Geochemistry, Tokyo University of Agriculture and Technology, Fuchu, Tokyo 183-8509, Japan

## Abstract

We investigated microplastics in the digestive tracts of 64 Japanese anchovy (*Engraulis japonicus*) sampled in Tokyo Bay. Plastic was detected in 49 out of 64 fish (77%), with 2.3 pieces on average and up to 15 pieces per individual. All of the plastics were identified by Fourier transform infrared spectroscopy. Most were polyethylene (52.0%) or polypropylene (43.3%). Most of the plastics were fragments (86.0%), but 7.3% were beads, some of which were microbeads, similar to those found in facial cleansers. Eighty percent of the plastics ranged in size from 150 μm to 1000 μm, smaller than the reported size range of floating microplastics on the sea surface, possibly because the subsurface foraging behavior of the anchovy reflected the different size distribution of plastics between surface waters and subsurface waters. *Engraulis* spp. are important food for many humans and other organisms around the world. Our observations further confirm that microplastics have infiltrated the marine ecosystem, and that humans may be exposed to them. Because microplastics retain hazardous chemicals, increase in fish chemical exposure by the ingested plastics is of concern. Such exposure should be studied and compared with that in the natural diet.

The plastic use continues to increase worldwide, and some waste plastics are released into the oceans^1^,^2^. Plastic debris is ubiquitous in the oceans around the world; it is estimated that at least 5.25 × 10^12^ plastic particles weighing 2.7 × 10^5^ t are currently floating at sea^3^. In particular, microplastics (defined as plastics <5 mm^4^) are the most common size fraction in seawater^3^,^5^,^6^. They are divided into primary and secondary microplastics by their sources. Primary microplastics are plastic particles originally manufactured at those sizes. Secondary microplastics are fragments generated by the breakdown of larger pieces. Fragmentation of plastics at sea occurs through photodegradation, physical impacts, and other processes, and results in the generation of a larger number of smaller particles^2^. Most microplastics in the marine environment are secondary^3^,^7^,^8^.

To the best of our knowledge, this is the first report that chemically identified microbeads, which are one of the major primary microplastics, in fish. Microbeads are spherical or amorphous particles used in personal care and cosmetic products^9^. They are made most commonly of polyethylene (PE), followed by nylon, polypropylene (PP), poly(methyl methacrylate) (PMMA), and poly(ethylene terephthalate) (PET)^10^. Microbeads are discarded down the drain and carried to sewage treatment plants (STPs). Although they are efficiently removed by settling during treatment, a small but significant proportion is discharged in final effluent^11^. In addition, combined sewer overflows introduce microbeads into receiving waters. After their discharge, microbeads stay in water bodies for a long time because of their non-biodegradable nature. Microbeads have been reported in surface waters^12^,^13^. The other primary microplastics include plastic resin pellets, industrial scrubbers, and plastic powder. The pellets are feedstock for consumer products, and are generally cylindrical or disk-shaped^14^. Industrial scrubbers include synthetic ‘sandblasting’ media made from polymers such as acrylic, polystyrene, melamine, and polyester^15^.

Marine plastics affect a wide range of species, from invertebrates to seabirds and whales^16^. Microplastics are bioavailable to many species^16^, and are easily ingested by planktivorous or smaller organisms^17^. Physical impacts such as injury or clogging of the digestive tract^18^ and impairment of feeding capacity have been observed^19^. Moreover, because microplastics contain hazardous chemicals sorbed from seawater as well as retained additives^20^, their toxicological impacts are of concern. An increasing number of reports document the ingestion of plastics, including microplastics, by fish species^21^,^22^,^23^,^24^,^25^,^26^. However, only a few studies^22^,^25^,^26^ reported polymer types. This information is essential to assessing the risks of chemicals associated with ingested plastics, because the composition and magnitude of the chemicals vary among polymer types; for example, PE absorbs more hydrophobic chemicals, such as polychlorinated biphenyls (PCBs), than other polymers^20^. In addition, specific additives are compounded into specific polymers; for example, phthalates in PVC or hexabromocyclododecane (HBCDD) in polystyrene^27^,^28^. The identification of polymer types is important also to identifying the sources of microplastics, as are detailed observations of shape and size; for example, microbeads are made mostly of PE, and some of them are spherical. In particular, the discrimination between primary and secondary microplastics is essential for source control. We investigated the plastics in Japanese anchovy (*Engraulis japonicus*) caught in Tokyo Bay, off the Tokyo metropolitan area (Fig. 1). The population in the drainage basin is 29 million, which accounts for one-fourth of the total population of Japan^29^. Tokyo Bay receives river water, sewage, industrial wastewater, and surface runoff from the city. We assumed that domestic and industrial activities are important sources of microplastics in Tokyo Bay. Sewage from a large proportion of the population in the catchment is treated by combined sewer systems, so untreated wastewater is occasionally discharged into the bay during heavy rain.

## Results

### Plastics in fish

We found plastics in the digestive tracts of 49 of the 64 anchovies (77%). Each fish had an average of 2.3 (±2.5) pieces of ingested microplastic, and from 0 to 15 pieces of debris. All of the plastics were photographed (Supplementary Table S1) and identified by FT-IR (Fig. 2). Among all 150 pieces found, there were 129 fragments (86.0%), 11 beads (7.3%), 8 filaments (5.3%), and 2 foams (1.3%) (Table 1, Fig. 3a). The beads comprised 6 spherical PE beads, 1 granular PE bead (an aggregation of 1 large sphere and some small spheres), 2 granular PP beads (shaped like a bunch of grapes), and 2 spherical white PS beads (Fig. 4). The longest length of the pieces ranged from 150 to 6830 μm (average 783 μm ± 1020) (Fig. 5), and the width ranged from 68 to 1880 μm (average 345 μm ± 272). The plastics consisted primarily of PE (52.0%) and PP (43.3%), followed by PS (2.0%), ethylene/propylene copolymer (2.0%), and ethylene/propylene/diene terpolymer (0.7%) (Fig. 3b). Most plastics were white (40.0%) or transparent (31.0%), with a lower presence of green (12.3%), yellow and yellowed (12.3%), black (2.6%), brown (1.3%), and pink (0.6%).

### Microbeads in personal care products

We investigated plastic particles in four brands of facial cleansers (labeled M1, M2, R1, S1) manufactured by three companies. All four products contained plastic particles, identified by FT-IR as PE, as stated in the list of ingredients. The size of all microbeads ranged from around 10 μm to 500 μm. M2 and S1 contained spherical microbeads of PE, with average diameters of 314 ± 120 μm (M2) and 188 ± 80 μm (S1). M1, M2, and R1 contained amorphous particles (irregular shapes, including thread-like) of PE, with average lengths of 247 ± 96 μm (M1), 295 ± 54 μm (M2), and 117 ± 58 μm (R1) (Fig. 6). The spherical microbeads in M2 were blue and transparent, and those in S1 were transparent. All of the amorphous particles were transparent or white.

## Discussion

The frequency of occurrence of plastics in the digestive tract of Japanese anchovy, at 77%, is one of the highest recorded in fishes^22^,^24^. Most of the ingested plastics were fragments, followed by beads and filaments derived from fishing gear, and most were PE or PP (Table 1, Fig. 3). This proportion of plastics is consistent with those in previous studies of plastic debris in surface seawater, which were dominated by fragments^3^,^7^,^8^ and by PE or PP^7^,^30^,^31^. The fragments have various surface features, such as sharp edges with cracks, rounded shapes with smooth surfaces, or degraded rough surfaces (Supplementary Table S1). Although we couldn’t identify the sources of the fragments, their appearance may relate to their origin or history of degradation in the environment. Further study for their source identification is needed. Among the major polymers, only PE and PP are less dense than seawater, and therefore they predominate in surface water^2^. Japanese anchovy are known to stay in pelagic shallow water at around 10 m depth^32^, and thus the proportion of ingested plastics reflects that of the water. The predominance of fragments has been also observed in fish from the North Pacific Central Gyre^21^ and in fish at a market in Indonesia^24^. On the other hand, fibers accounted for 68.3% of plastics in fish from the English Channel, which were identified as rayon, polyamide, or polyester^22^. Fibers were also predominant in fish from the USA^24^. This difference may be due to regional source differences or feeding habits of fish species; further studies are needed.

To the best of our knowledge, our study provides the first evidence of the ingestion of microbeads, suspected of being derived from personal care products, by fish. Beads accounted for 7.3% of the plastics in the anchovies, and most were PE (Table 1, Fig. 4). These artificial shapes strongly indicate that they are manufactured as micro-sized products. Over 90% of products with microbeads list polyethylene in the ingredients, and the others include PP (calculated from lists provided by Beat the Microbead^10^). Our results confirm the presence of PE particles in four brands of facial cleansers popular on the Japanese market, and spherical microbeads in two of them (Fig. 6). The spherical PE beads detected in the fish were similar in size and appearance to those in the facial cleansers (Figs 4 and 6). One of PE beads in the anchovy was shaped like a single large sphere aggregated with some small spheres (Fig. 4e). Because it was reported that some facial cleansers contain granular PE microbeads as well as spherical ones^33^, the non-spherical PE bead in the anchovy is likely to be originated from such granular microbeads in the personal care products. Both PP beads we found in anchovy were granular and were shaped like a bunch of grapes (Fig. 4j,k), and may also be derived from personal care products. However, personal care products that contain PP beads are uncommon on the Japanese market based on our survey. It is also reported that only a few percent of products contains PP beads while PE accounts for >90 percent on the world market^10^. The detection frequency of PP beads relative to PE beads seems higher than expected from the current market share, so PP beads in the fish may be derived from other sources. Finally, the white polystyrene beads that we found accorded with the shape and size of pre-expanded polystyrene beads, which are spherical and measure 0.1 to 2 mm^34^.

Eriksen *et al*.^12^ found many multi-colored spherical polymers of <1 mm in surface waters of the Laurentian Great Lakes of North America^12^. Mani *et al*.^13^ detected opaque spherules identified as PE on the surface of the Rhine River^13^. These studies identified them as microbeads used in consumer products such as facial cleansers^12^,^13^. Some studies found spherical microplastics in some organisms, such as in commercial bivalves from China^35^ and in zooplankton samples from the English Channel^36^, but the polymer types were not reported. It is difficult to discuss sources without information on polymer types, because there are other sources of spherical microplastics than personal care products, as indicated by the presence of many non-PE spherical beads (such as acrylic, polyurethane, and polyester copolymers) on the sea surface in a South Korean bay^37^.

Microbeads in personal care and cosmetic products are discarded down the drain after use and go through several treatment processes. Although 95% to 99.9% of them are removed by settling at STPs, the remainder are discharged with effluent and end up in the aquatic havitats^11^. More importantly, combined sewer overflows can brings large amounts of untreated wastewater containing microbeads to coastal waters; in Tokyo, about half of the population in the catchment (29 million) is served by combined sewer systems^38^. When heavy rain (generally > 5 mm) falls, sewer overflows occur and could flush microbeads out. On the basis of the capacity of STPs and precipitation patterns in Tokyo, overflows occur around 50 times a year^39^, implying that ~7% ([50 days/365 days] × 0.5) of microbeads used in the catchment are discharged into aquatic environments without settling during treatment.

We found spherical microbeads in fish stomachs. As we could not distinguish amorphous microbeads (Fig. 6) from degraded microplastic fragments, the “fragments” may include amorphous microbeads, and the distribution of microbeads may be underestimated.

The size distribution of microplastics in the anchovies was different from those reported in surface seawaters around Japan^6^. Over 80% was <1000 μm and more than half was <500 μm in size (Fig. 5), although anchovy can ingest prey from several tens of μm^40^ to >5 mm (such as zooplankton we found in the gut of some fishes). In the surface waters around Japan, however, microplastics (<5 mm) larger than 1 mm dominate smaller ones, which account for around 20% of all microplastics, and the size distribution peaked around 1 mm^6^. Although the number of smaller microplastics in seawater samples is underestimated owing to sampling bias due to net size (350-μm mesh), the difference in the size distribution of microplastics between the anchovies and surface seawaters is clear only in the comparison of microplastics of >400 μm. This difference is probably due to the feeding characteristics of Japanese anchovy, which are pelagic filter feeders and ingest suspended particles in subsurface waters. Because smaller plastic particles have lower rise velocities, smaller plastics (0.5–1.0 mm) were more abundant in subsurface water than in surface water in the North Atlantic Gyre^5^. If the same vertical profile of microplastics occurs in Tokyo Bay and smaller plastics are more abundant than larger plastics in the subsurface water, by feeding in subsurface water the anchovies could accumulate smaller microplastics in their digestive tract. More studies are necessary to understand the vertical profiles of microplastics of various size ranges.

Japanese anchovy is widely distributed around Japan and is a common food in Japan. It is one of the most caught fish species in Japan^41^, and is typically eaten without removal of the digestive tract. Nine species of *Engraulis* are distributed in coastal waters around the world and are ecologically important because of their huge biomass and their central role in the diet of many fish, birds, and marine mammals^42^. Our observations further confirm that microplastics have infiltrated marine ecosystems globally, and humans are now exposed to them, although plastics within the observed size range (0.2–5 mm) would be excreted if we ate contaminated anchovies. But plastics in the marine environment contain various hazardous chemicals, both additives compounded during manufacture and hydrophobic chemicals adsorbed from seawater^20^. The ingestion of microplastics by anchovies may increase the body burden of the hazardous chemicals to both anchovies and humans. Regarding chemicals sorbed to microplastics, their transfer and accumulation in fish tissue upon ingestion were demonstrated by laboratory exposure experiments^43^,^44^. On the other hand, anchovies and humans are exposed to hazardous chemicals through natural prey, too. The low number of microplastics that we found in the anchovies (~2 pieces per fish on average) suggests that chemical exposure through the ingested microplastics is minor compared with that from the natural diet. However, inputs of plastics into the oceans and number of ingested plastics in fish will continue to increase if no action is taken, and exposure of additive-derived chemicals may become more important in future.

Once plastics are discharged into the marine environment, they are difficult to recover, especially if they are small. Therefore, reducing the amount discharged from land to the oceans is the first priority. To the best of our knowledge, this is the first study to identify and confirm microbeads in fish by examining polymer types and shapes. The USA and some other countries now regulate microbeads from rinse-off personal care products. Japanese cosmetics companies have just started voluntary elimination of microbeads from their products. While laudable, it is important to note that these regulations do not cover all products containing microbeads and other forms of microplastics. Global controls on microbeads should be considered, with restrictions on a wider range of products. More importantly, however, plastic fragments should be regulated with first priority, because a majority of the microplastics in the fish were fragments. For the effective regulation, identification of the origins or original products of individual fragments are necessary. Their appearance (shape) and color can be connected to the original products, though both, especially the former, are altered by photo-oxidation. Identification of additive chemicals specific to specific original products or their usage may help source identification. Control of plastic products which can be easily fragmented is important to solving pollution of the marine environment by plastics.

## Methods

### Sampling and processing

**Figure 1:**
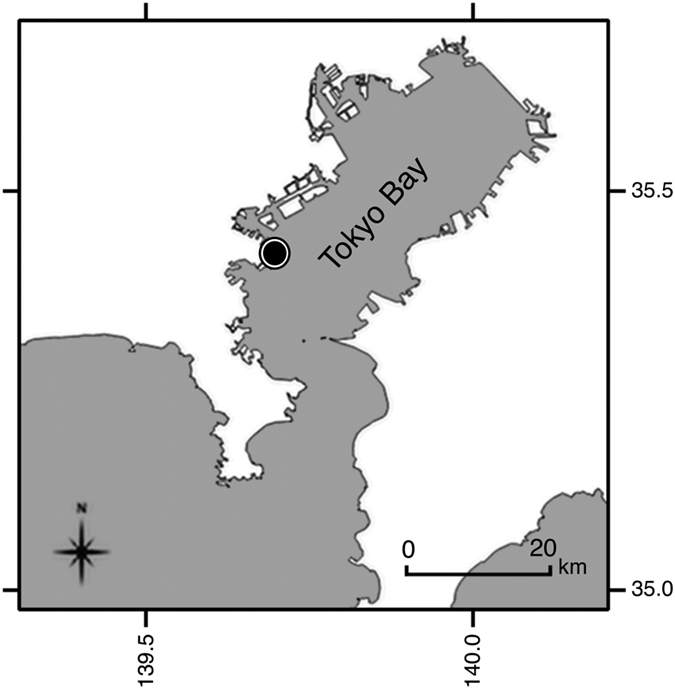
Sampling location in Tokyo Bay. Sixty-four Japanese anchovy (*Engraulis japonicus*) were caught on 23 August 2015. Map created using QGIS^47^ and data provided by the Geospatial Information Authority of Japan^48^.

**Figure 2:**
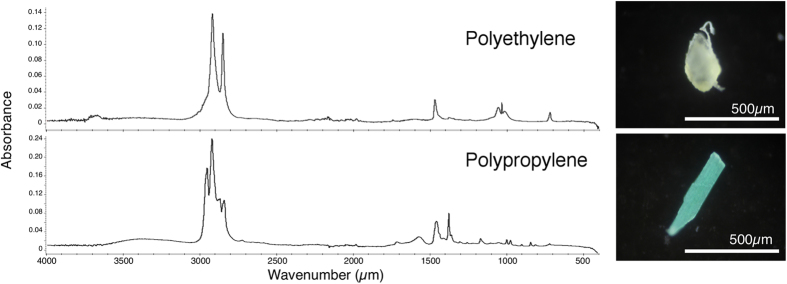
Spectra of FT-IR analysis and photographs of plastic fragments. All suspected plastics were identified by FT-IR and photographed. Two examples are shown.

**Figure 3:**
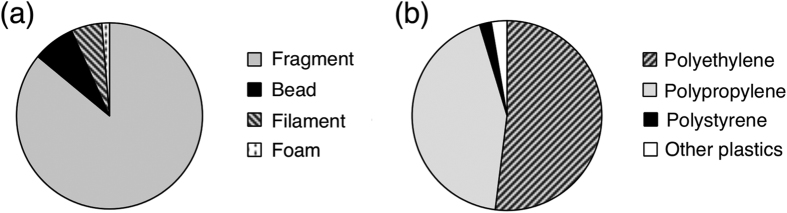
Types of plastics recovered from digestive tracts of Japanese anchovy (*Engraulis japonicus*). (**a**) Percentage by shape. (**b**) Percentage by polymer.

**Figure 4:**
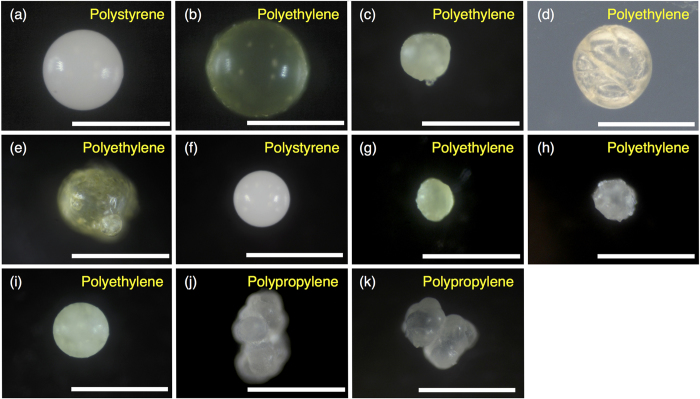
Photographs of microbeads ingested by Japanese anchovy (*Engraulis japonicus*). Scale bar represents 500 μm. All photographs were taken in reflected light, but in the case of *d*, only photographs taken with transmitted light are available.

**Figure 5:**
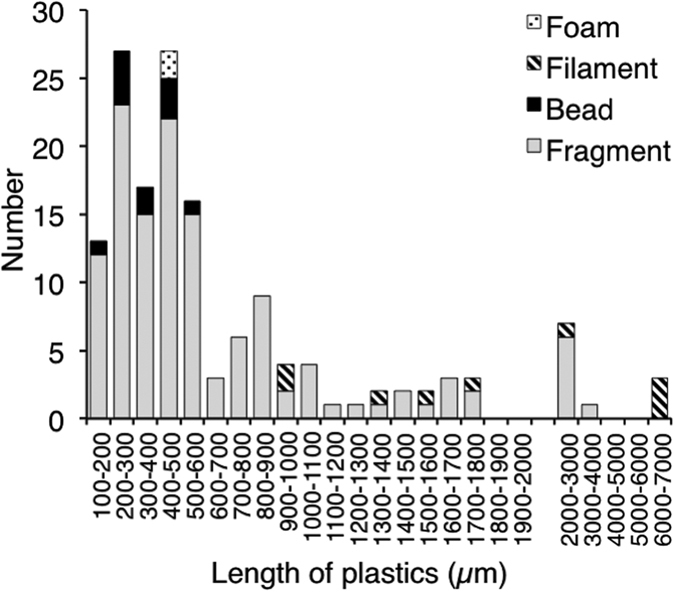
Size distribution of plastics in Japanese anchovy (*Engraulis japonicus*).

**Figure 6:**
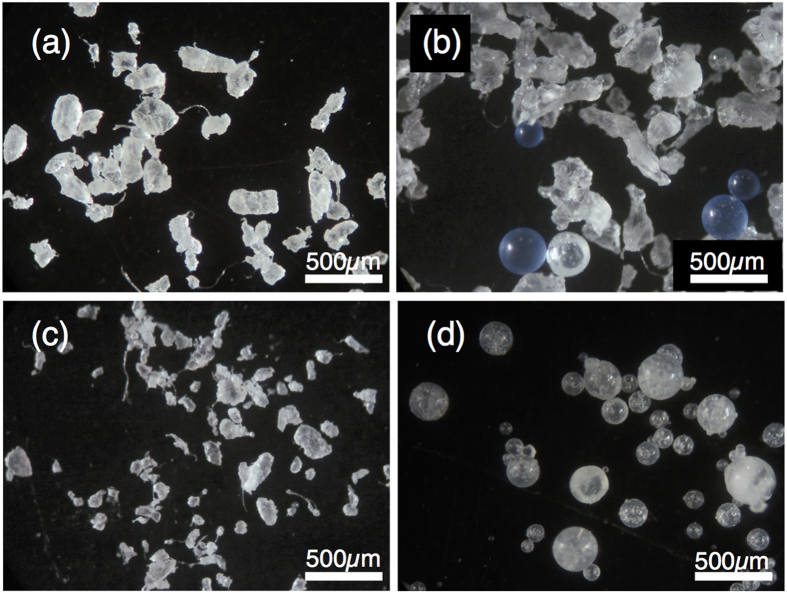
Photographs of polyethylene microbeads in four brands of facial cleansers. (**a**) Brand M1, irregular shapes. (**b**) Brand M2, blue and transparent spheres and irregular shapes. (**c**) Brand R1, irregular shapes. (**d**) Brand S1, transparent spheres.

The Japanese anchovy (*Engraulis japonicus*) were caught by fishing using Sabiki rigs from a pier in Tokyo Bay (35°25′43″N 139°41′15″E) (Fig. 1) from 07:00 to 14:00 on 23 August 2015. The depth of the water there was around 15 to 20 m, and the fish were caught at a range of 5 to 10 m from the surface. We collected 64 anchovy. The fish were put in iced water and dissected at the laboratory the same day. After measurement of their body length (112.5 mm ± 6.4 mm), we removed the whole of the digestive tract (from top of the esophagus to the anus) and put it into a 10-mL glass vial that had been baked for 4 h at 550 °C in advance. Each vial then received 7–8 mL (>3× the volume of the gut) of 10% KOH solution to digest organic material^23^,^24^. The vials were incubated at 40 °C for 10 days, during which digestion was observed to be completed in 3 to 4 days. Each vial was then shaken around 20 times to break up the mass of indigestible materials such as shells of zooplankton, and all floating material was collected in another vial. Pieces larger than 200 μm were clearly visible. The precipitate that remained in the vial was put on a glass Petri dish and examined under a microscope, but no particles not resembling natural prey were observed. Because our target fish (anchovy) is commonly caught by recreational-fishing and eaten by people and our procedure of fishing and dissection is exactly same as what people fish and cook it by, our procedure has no ethical problem. Our procedure of measurement of microplastics in the digestive tract does not conflict with ethical rules for animal experiment of our university.

### Classification and identification of plastics

All floating items suspected to be plastic polymers were photographed individually (Supplementary Table S1), and the color and shape were recorded. The definition of the shape of microplastics is as follows: fragments: particles produced by fragmentation of larger materials, beads: particles manufactured as micro-sized products, either spherical or an aggregate of spheres, pellets: granules manufactured as a raw material of larger plastic products, generally in the size range of 2–5 mm with shape of a cylinder or a disk, foams: foams made from polymer, films: soft fragments of thin polymers derived from plastic bags or wrapping paper and so on, sheets: hard fragments of thin polymers, filaments: thread-like polymers produced by fragmentation of ropes or lines used in fishing, >50 μm, and fibers: thread-like polymers derived from textiles, including clothing and furnishings, ≤50 μm. The 50-μm threshold for fibers was chosen because typical textile fibers have a diameter of 10 to 20 μm (up to 50 μm)^45^, and the diameter of monofilaments used in fishing ropes or lines is larger than several hundred μm^46^.

We analyzed all of the pieces of suspected plastic (*n* = 173) by Fourier transform infrared (FT-IR) spectroscopy (Nicolet iS10, Thermo Scientific) to identify polymer types. The IR absorbance from 450 to 4000 cm^−1^ was compared with spectra in the software database, with a similarity threshold of >70%. Twelve particles were identified as having a natural origin, 11 could not be identified, and the others (i.e., 150 pieces) were identified as synthetic polymers.

To avoid contamination, we kept samples sealed in a vial or Petri dish at all times except when picking out the suspected plastics. A procedural blank analysis found no plastics. To estimate airborne contamination, we put Petri dishes (total 17 000 mm^2^) on a table near the work bench for 1 day to collect airborne particles. We collected 3 polymer fibers (PET, polyamide [similarity < 70%], and polyolefin [similarity < 70%]), and more than 10 cotton or wood fibers, but no other fibers. This means that procedural contamination did not significantly affect the results in the present study.

### Microbeads in personal care and cosmetic products

We examined plastics in personal care products to determine the features of the plastic beads used in them. We bought four major brands of facial cleansers that listed polyethylene as an ingredient. They were labeled M1, M2, R1, and S1. We mixed several grams of product in distilled water and identified floating solid particles by FT-IR.

## Additional Information

**How to cite this article**: Tanaka, K. and Takada, H. Microplastic fragments and microbeads in digestive tracts of planktivorous fish from urban coastal waters. *Sci. Rep*. **6**, 34351; doi: 10.1038/srep34351 (2016).

## Supplementary Material

Supplementary Information

## Figures and Tables

**Table 1:** Total number of plastic pieces in the digestive tract of Japanese anchovy (*Engraulis japonicus*) by shape and polymer.

	Fragment	Bead	Filament	Foam	TOTAL
PE	70	7	1		78
PP	54	2	7	2	65
PS	1	2			3
Others	4				4
TOTAL	129	11	8	2	150

PE, polyethylene; PP, polypropylene; E/P, ethylene/propylene copolymer; PS, polystyrene. “Others” include ethylene/propylene copolymer and ethylene/propylene/diene terpolymer.
