# Supplementary material for: Microplastic fragments and microbeads in digestive tracts of planktivorous fish from urban coastal waters
Source: Sci Rep. 2016 Sep 30;6:34351. doi: 10.1038/srep34351 (PMC5043373; doi:10.1038/srep34351)
Supplement: Supplementary Information [file srep34351-s1.pdf]

Supplementary Information

Microplastic fragments and microbeads in digestive tracts of planktivorous fish from urban coastal waters

Kosuke Tanaka[a], *Hideshige Takada*[a]*

[a] *Laboratory of Organic Geochemistry (LOG), Tokyo University of Agriculture and Technology, Fuchu, Tokyo 183-8509, Japan*

*Corresponding Author: shige@cc.tuat.ac.jp

Page 1 – 8

Supplementary Table S1

Supplementary Table S1. Photographs of plastics ingested by Japanese anchovy (*Engraulis japonicus*).
Scale bar represents 500 µm. PE, polyethylene; PP, polypropylene; E/P, ethylene/propylene copolymer; PS, polystyrene; E/P, ethylene/propylene copolymer; E/P/D, ethylene/propylene/diene terpolymer; Fr, fragment; B, bead; Fl, filament; Fo, foam.

Fish ID	Photographs of plastics		
#1	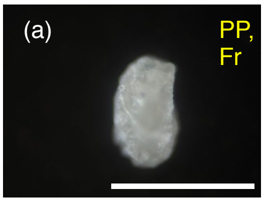		
#2	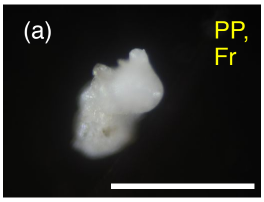	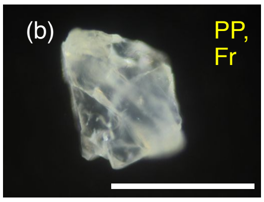	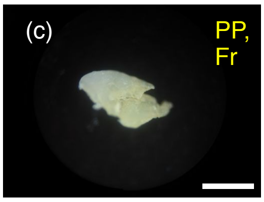
#4	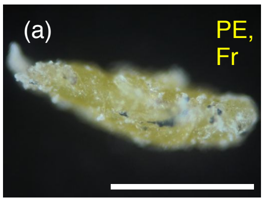	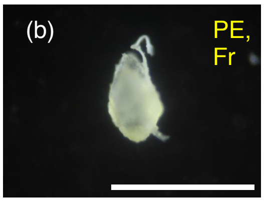	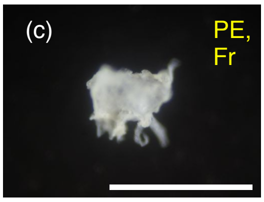
#5	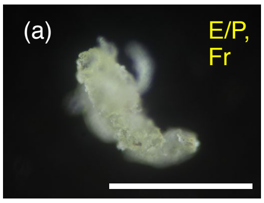	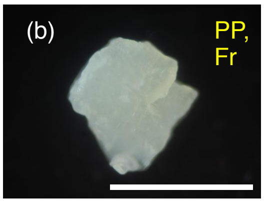	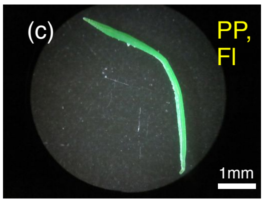
#6	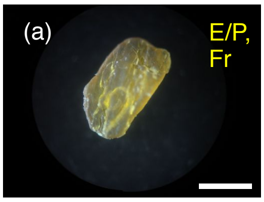		
#7	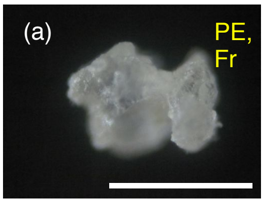		
#8	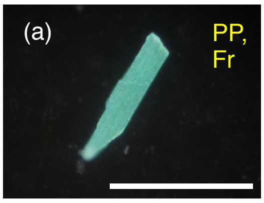		
#9	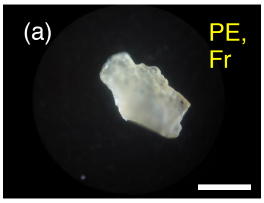		

(continued)

Fish ID	Photographs of plastics
#11	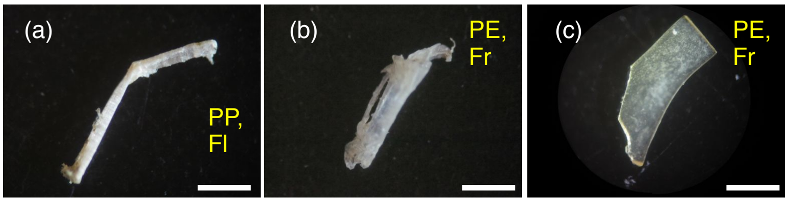
#12	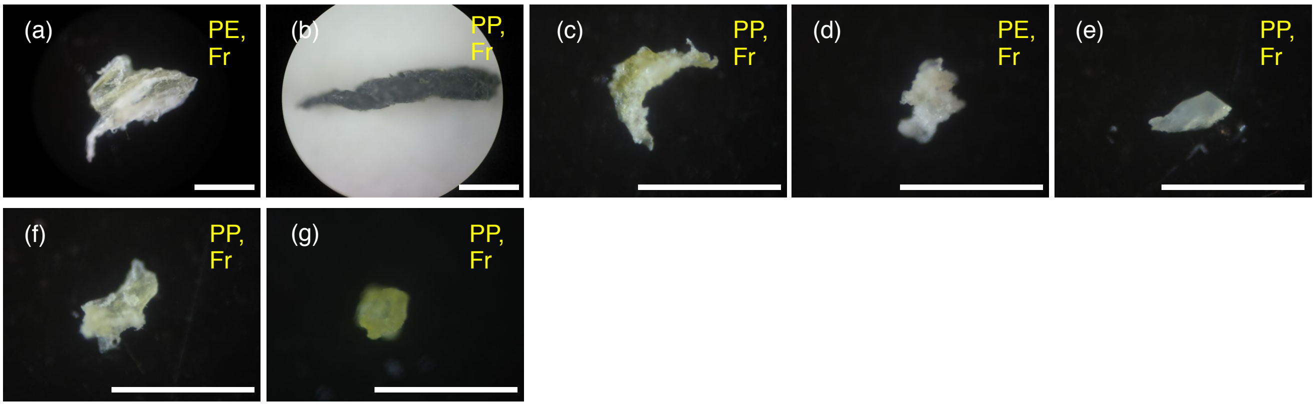
#13	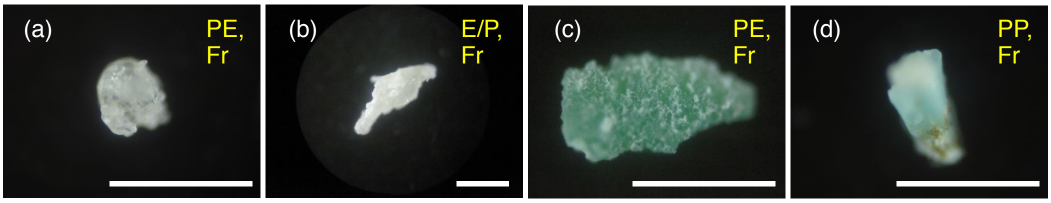
#15	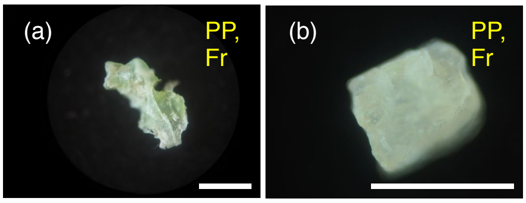
#16	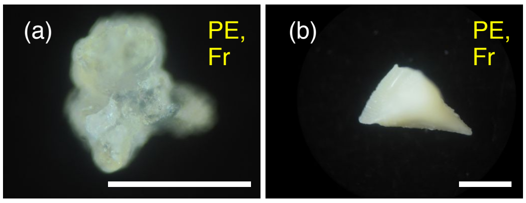
#17	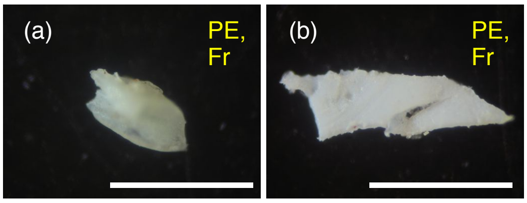
#18	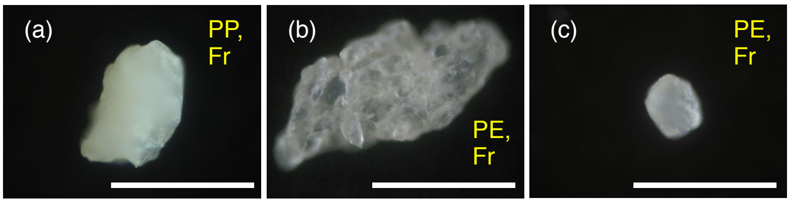

(continued)

Fish ID	Photographs of plastics
#19	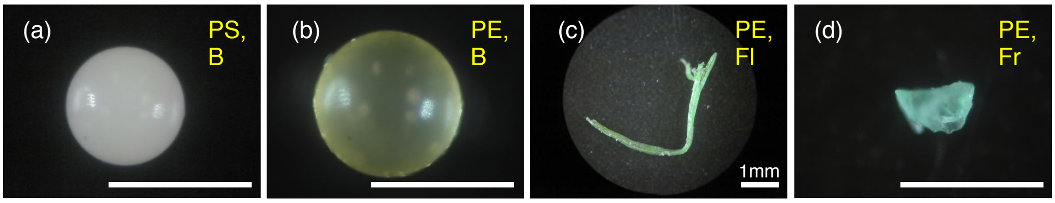
#20	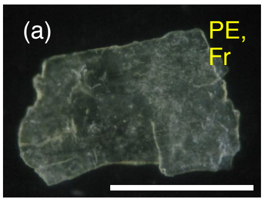
#21	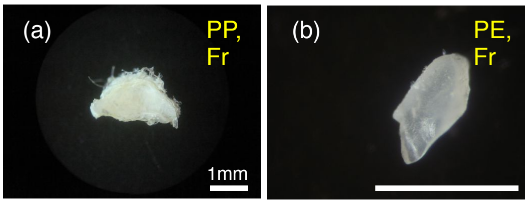
#22	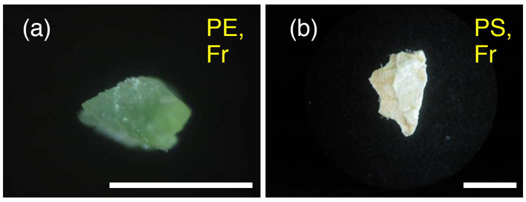
#24	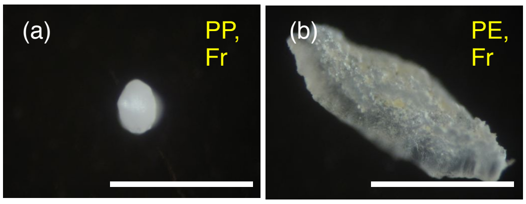
#25	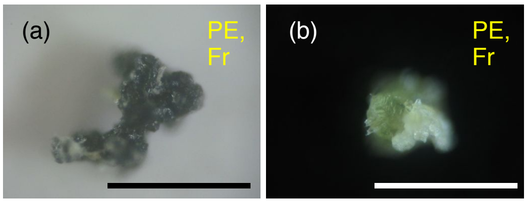
#27	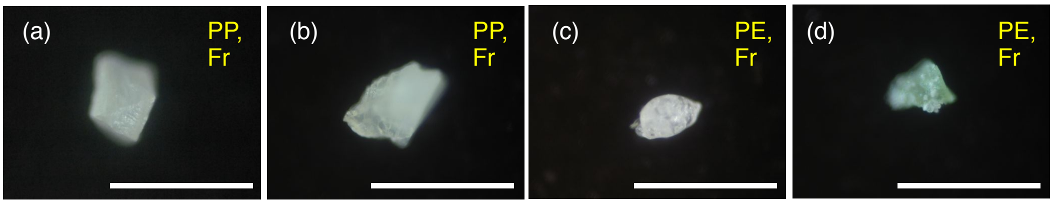
#28	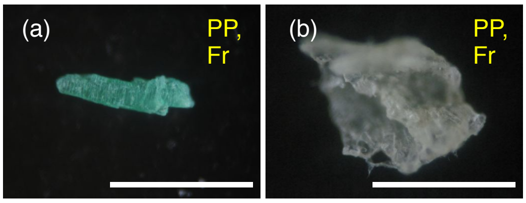

(continued)

Fish ID	Photographs of plastics

#29

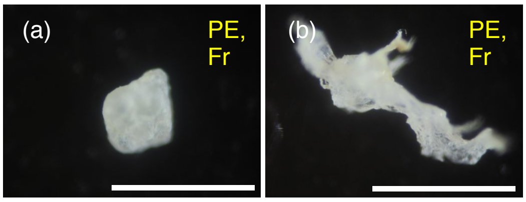

#34

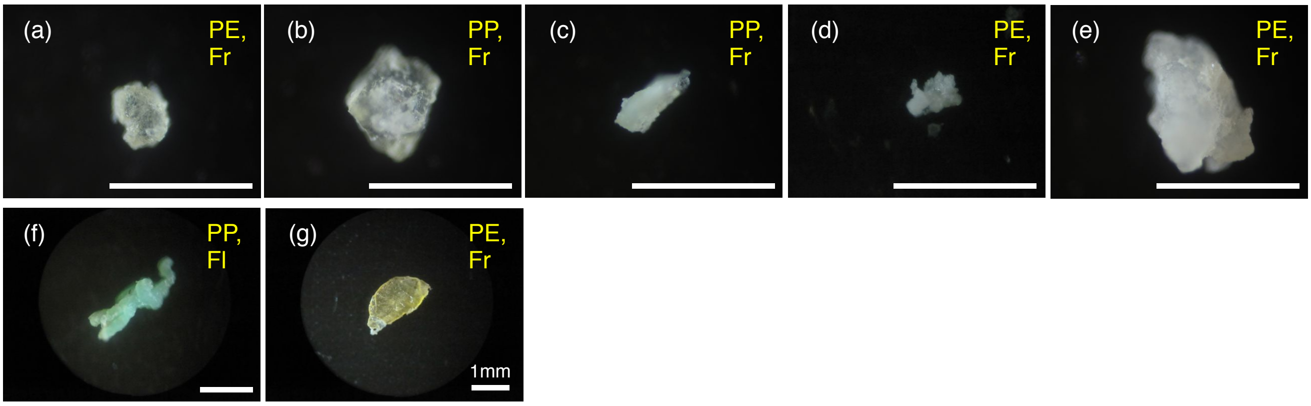

#35

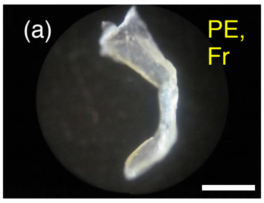

#37

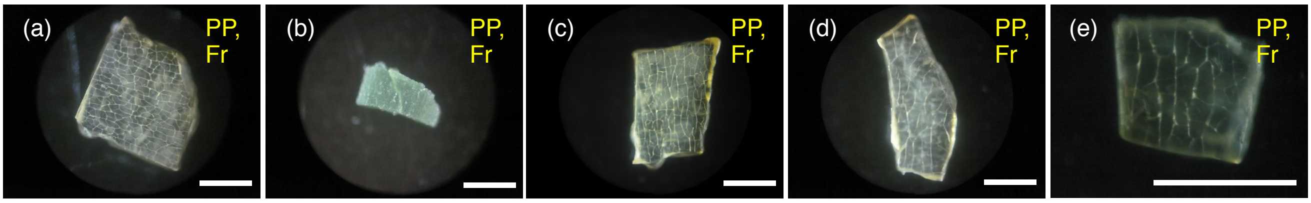

#38

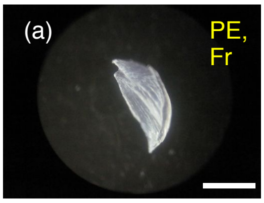

#39

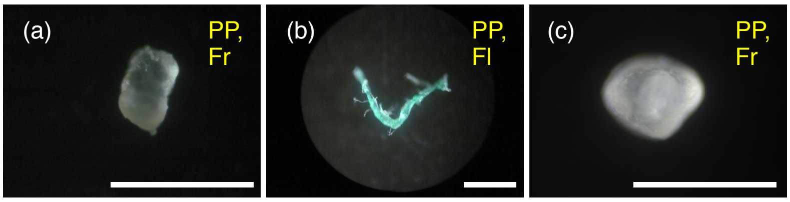

#40

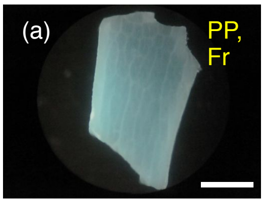

(continued)

Fish ID	Photographs of plastics
#41	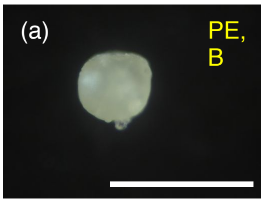
#42	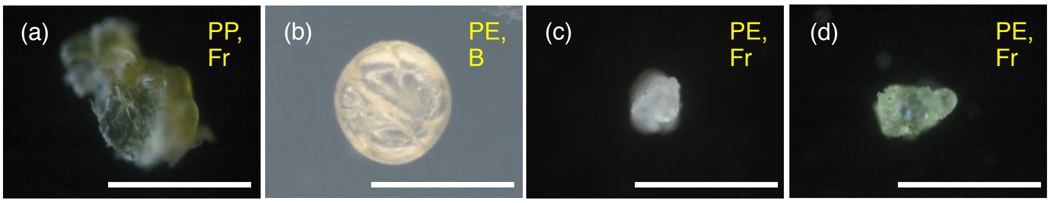
#43	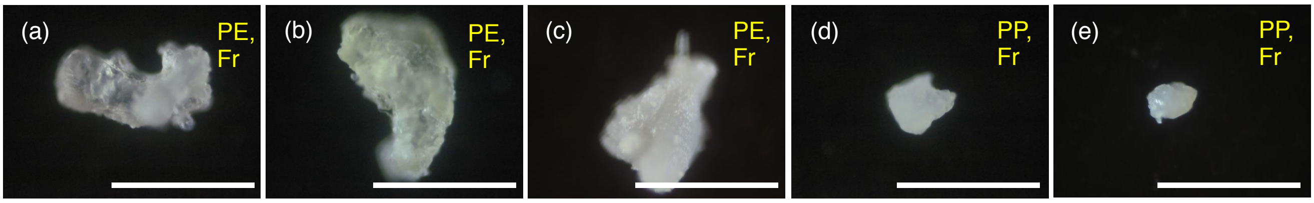
#44	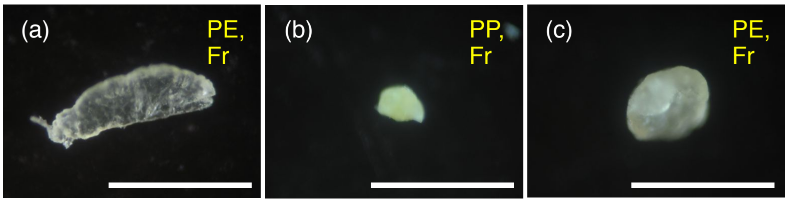
#46	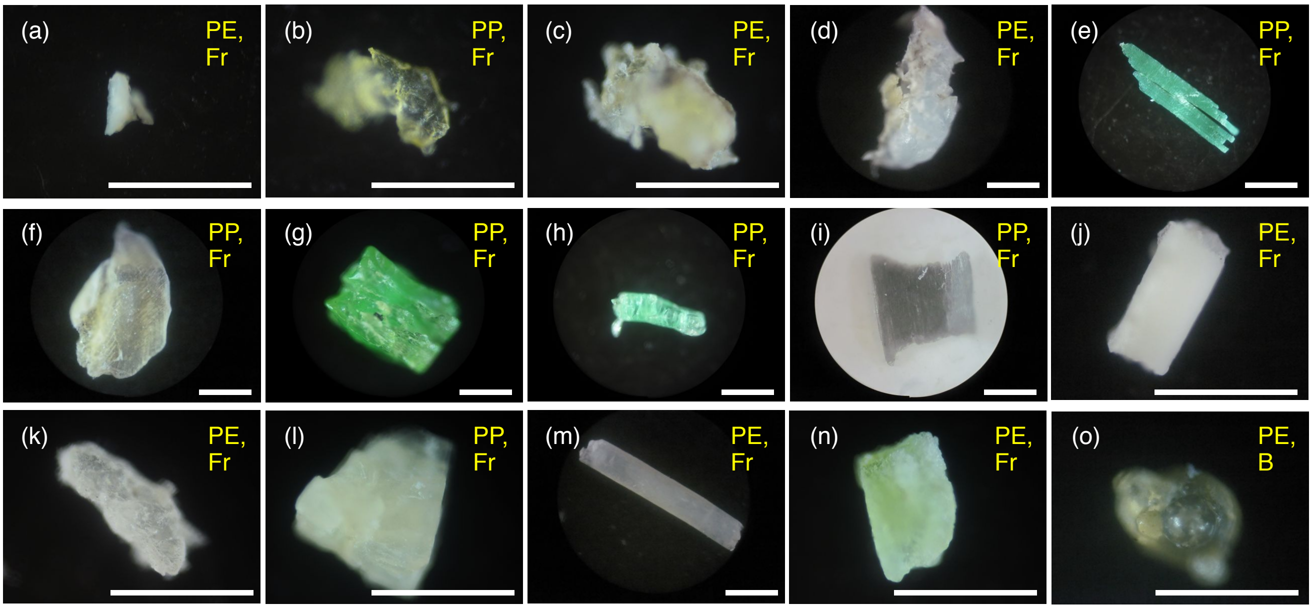
#47	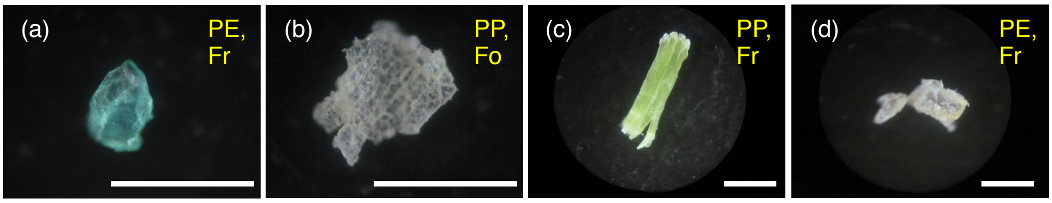

(continued)

Fish ID	Photographs of plastics
#49	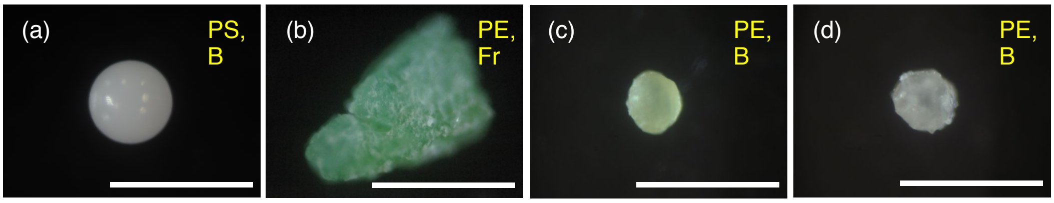
#50	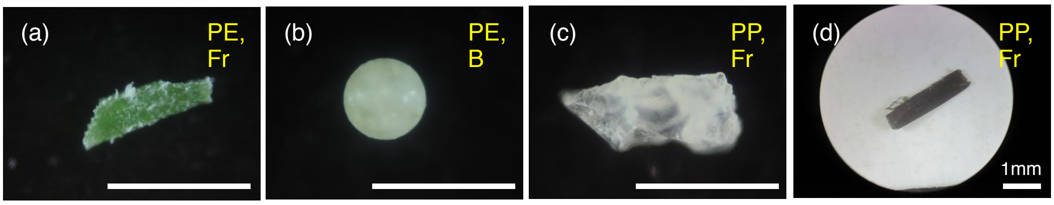
#51	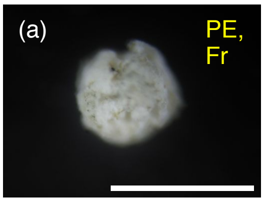
#52	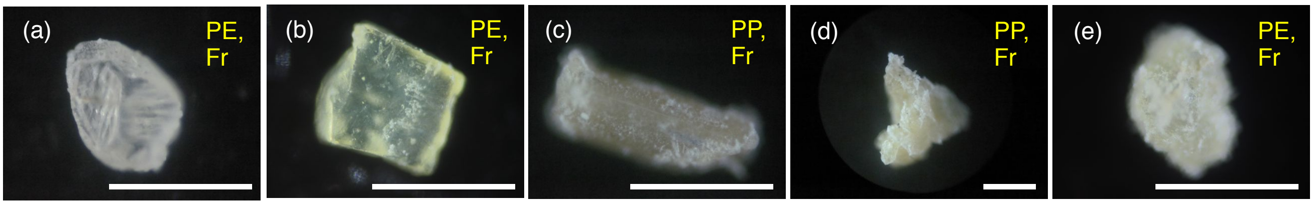
	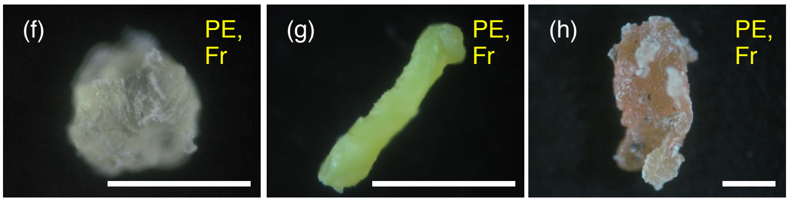
#53	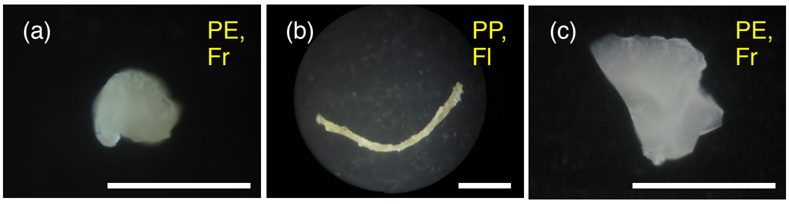
#56	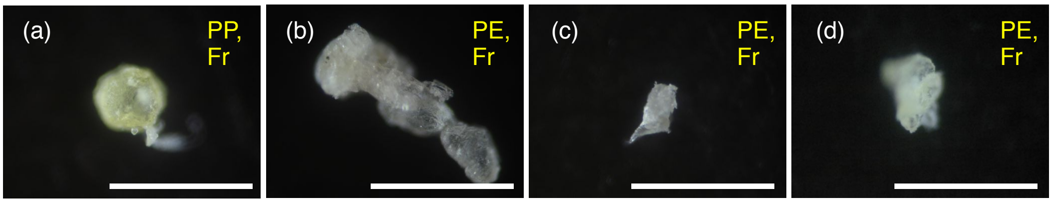
#57	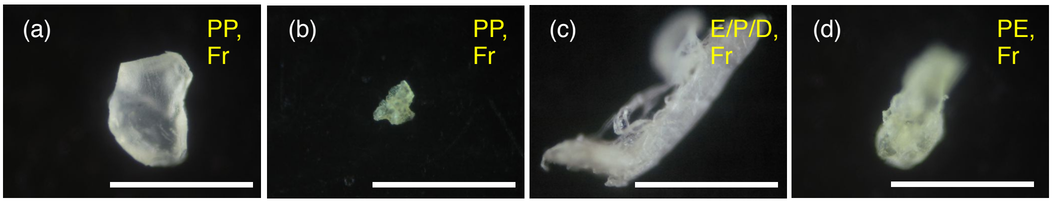

(continued)

Fish ID	Photographs of plastics
#58	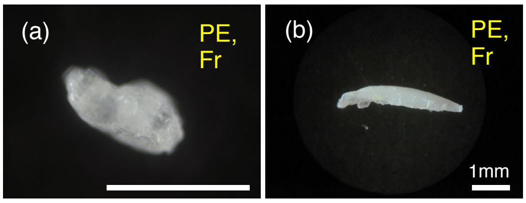
#60	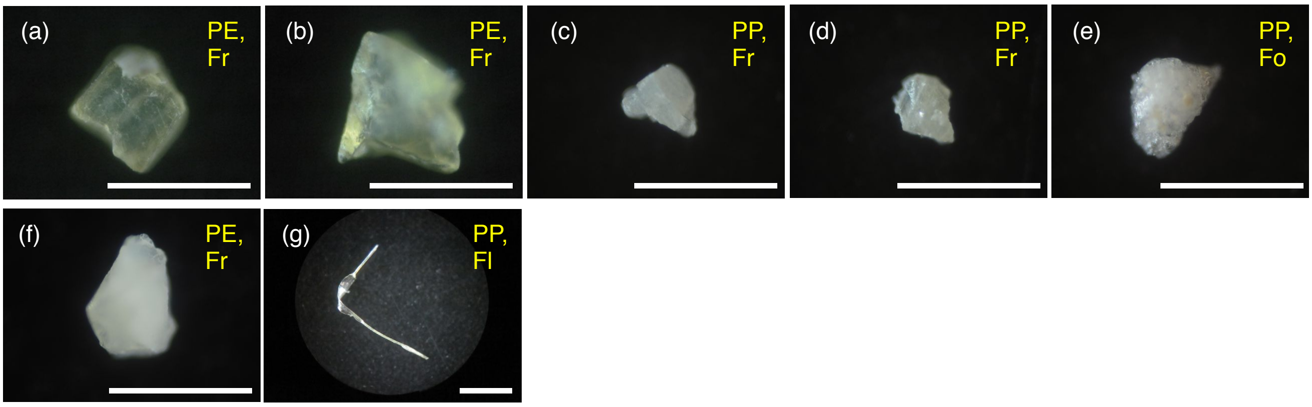
#61	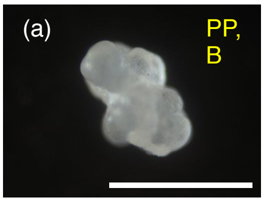
#62	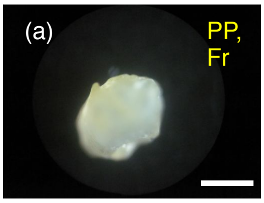
#63	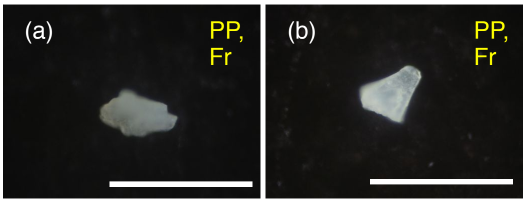
#64	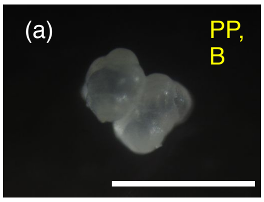
